# Factors Influencing *Legionella* Contamination of Domestic Household Showers

**DOI:** 10.3390/pathogens8010027

**Published:** 2019-02-26

**Authors:** Deanna Hayes-Phillips, Richard Bentham, Kirstin Ross, Harriet Whiley

**Affiliations:** College of Science and Engineering, Flinders University, GPO Box 2100, Adelaide 5001, Australia; haye0119@flinders.edu.au (D.H.-P.); Richard.Bentham@flinders.edu.au (R.B.); Kirstin.Ross@flinders.edu.au (K.R.)

**Keywords:** *Legionella pneumophila*, Legionnaires’ disease, opportunistic pathogens, potable water, water quality, public health

## Abstract

Legionnaires’ disease is a potentially fatal pneumonia like infection caused by inhalation or aspiration of water particles contaminated with pathogenic *Legionella* spp. Household showers have been identified as a potential source of sporadic, community-acquired Legionnaires’ disease. This study used qPCR to enumerate *Legionella* spp. and *Legionella pneumophila* in water samples collected from domestic showers across metropolitan Adelaide, South Australia. A survey was used to identify risk factors associated with contamination and to examine awareness of *Legionella* control in the home. The hot water temperature was also measured. A total of 74.6% (50/68) and 64.2% (43/68) showers were positive for *Legionella* spp. and *L. pneumophila*, respectively. Statistically significant associations were found between *Legionella* spp. concentration and maximum hot water temperature (*p* = 0.000), frequency of shower use (*p* = 0.000) and age of house (*p* = 0.037). Lower *Legionella* spp. concentrations were associated with higher hot water temperatures, showers used at least every week and houses less than 5 years old. However, examination of risk factors associated with *L. pneumophila* found that there were no statistically significant associations (*p* > 0.05) with *L. pneumophila* concentrations and temperature, type of hot water system, age of system, age of house or frequency of use. This study demonstrated that domestic showers were frequently colonized by *Legionella* spp. and *L. pneumophila* and should be considered a potential source of sporadic Legionnaires’ disease. Increasing hot water temperature and running showers every week to enable water sitting in pipes to be replenished by the municipal water supply were identified as strategies to reduce the risk of *Legionella* in showers. The lack of public awareness in this study identified the need for public health campaigns to inform vulnerable populations of the steps they can take to reduce the risk of *Legionella* contamination and exposure.

## 1. Introduction

Legionellae are opportunistic pathogens that have been identified as a major public health concern with incidences of Legionellosis continuing to rise [1]. The two clinical illnesses caused by *Legionella* spp. are Legionnaires’ disease, a potentially fatal pneumonia; and Pontiac fever, a milder form that mimics the symptoms of influenza [2]. The *Legionella* species reside ubiquitously in built water systems favoring temperatures between 20 and 45 °C. Transmission occurs through inhalation or aspiration of aerosolized *Legionella* spp. contaminated water particles [3]. Cooling towers, warm water systems and spa systems are well-documented sources of exposure and outbreaks of disease [4]. As a result, control and prevention measures have been established. However, little is documented on sporadic *Legionella* cases where the cause of infection is often not identified [5]. Household showers provide ideal environments for *Legionella* proliferation and exposure, and have often been proposed as a source of sporadic, community-acquired legionellosis [6].

Shower exposure as a potential route of transmission is a major concern for immunocompromised and elderly people that reside within the community as they are at the greatest risk of acquiring infection [7]. This is particularly significant given our increasing aging population, with global estimates suggesting that by 2025 25% of the world’s population will be over 60 years old [8].

The global demand for aged care has been rapidly increasing. In Australia, it is estimated that from 2010 to 2050 the Australian governments’ spending on aged care will double relative to national income [9]. There is also an increase in the number of individuals wishing to remain in their own homes and part of the community for longer. From June 2017 to June 2018, there was a 28.6% increase in the number of home care packages provided by the Australian government to individuals wishing to remain living independently in their own homes [10]. Consequently, there is an increasing number of elderly individuals and vulnerable people residing within their own homes, with showers that are not regulated for the control of *Legionella.*

Exposure of people over 65 years to *Legionella* in their homes raises concern about possible public health implications. The aim of this study was to investigate the presence of *Legionella* spp. and *Legionella pneumophila* in domestic showers and identify factors that may increase the likelihood of contamination. Additionally, the general public’s awareness of *Legionella* control within the home was investigated. Increased awareness may help to reduce the risk of *Legionella* exposure in the domestic environment, protecting our vulnerable populations.

## 2. Results

**Figure 1:**
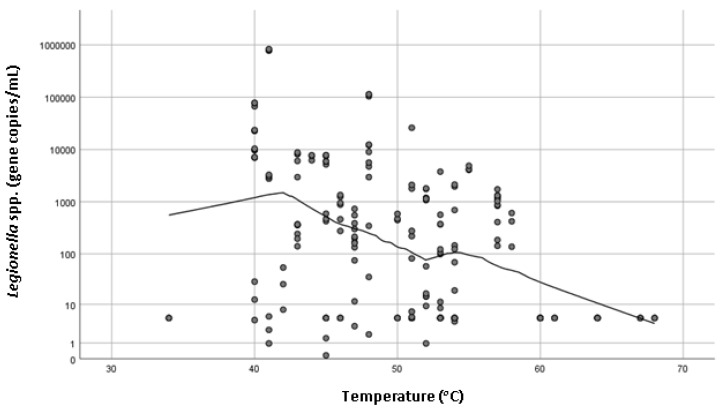
Scatter plot showing the set hot water temperature (°C) and *Legionella* spp. (copies/mL) on log_10_ scale with loess line of fit.

**Figure 2:**
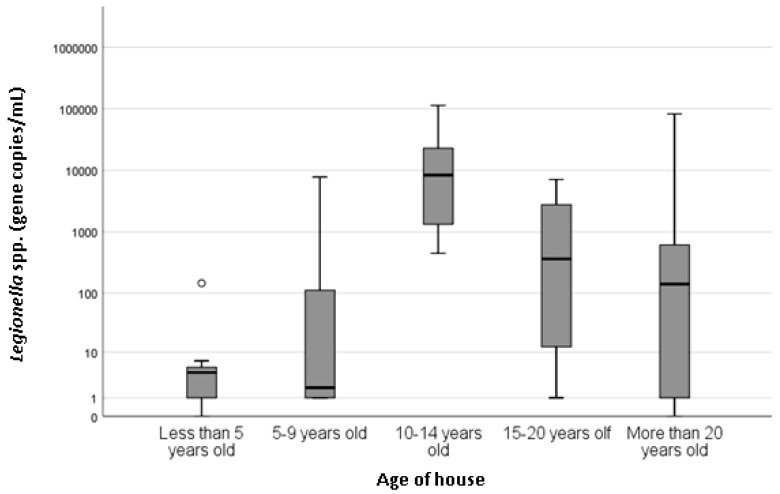
Boxplot showing mean concentration of *Legionella* spp. (copies/mL) on a log_10_ scale by the age of the house (determined by survey responses).

**Figure 3:**
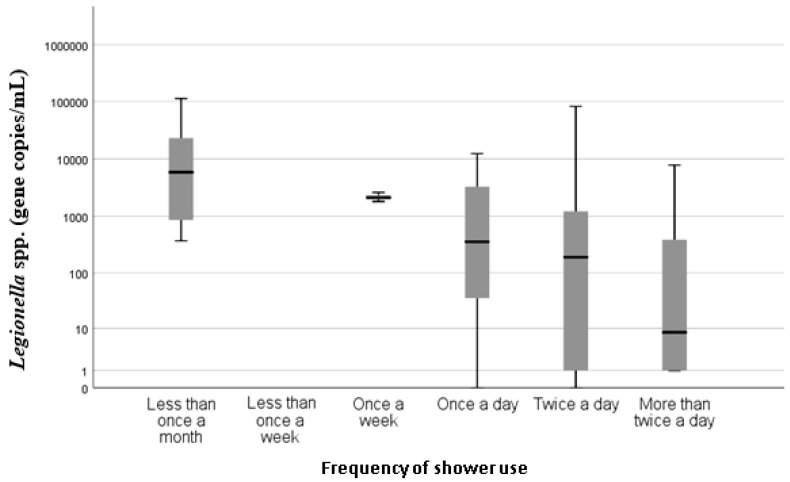
Boxplot showing mean concentration of *Legionella* spp. (copies/mL) on a log_10_ scale by the frequency of shower use (determined by survey responses).

Of the 68 shower samples enumerated using qPCR, 74.6% (n = 50) were positive for *Legionella* spp. and 64.2% (n = 43) were positive for *L. pneumophila*. The concentration of those positive samples ranged from 2.5 copies/mL to 110,000 copies/mL and the mean concentration was 7603 copies/mL and 4295 copies/mL for *Legionella* spp. and *L. pneumophila*, respectively. No statistically significant association (*p* > 0.05) with temperature, type of hot water system, age of system, age of house or frequency of use and *L. pneumophila* concentrations were observed. However, there was a statistically significant association (*p* = 0.000) between *Legionella* spp. concentration and the temperature of hot water measured at the outlet (Figure 1). Recorded hot water temperatures ranged from 34 to 68 °C, and the mean was 50 °C. Property age ranged between less than 5 years old to more than 20 years old. There was a statistically significant association with the age of the house (*p* = 0.037) and *Legionella* spp. concentration (Figure 2), with houses less than 5 years old associated with the lowest *Legionella* spp. concentration. There was also a statistically significant (*p* = 0.000) association between the frequencies of shower usage and *Legionella* spp. concentration, with showers used less than once a month having higher concentrations of *Legionella* spp. compared with showers that were used once a week or more frequently (Figure 3).

There was no statistically significant association between *Legionella* spp. and other household variables measured. The majority of hot water systems were gas (73.8%), followed by electric (15.4%) and solar (4.6%) (6.2% of participants did not know). Hot water storage was also examined, 58% were reported to be instantaneous, 28% stored in a tank and 14% of the respondents did not know. The number of showers in the 50 homes surveyed ranged from 1 to 4, with a mean of 1.72 showers per home. Age of hot water system was also investigated, 34% of hot water units were less than 5 years old, 12% were 5–9 years old, 14% were 10–14 years old, 4% were 15–20 years old, 10% were more than 20 years old and 38% did not know how old their hot water system was.

When survey participants were asked if they knew what the temperature of the hot water system was set at, 70% of participants did not know, indicating the lack of awareness of bacterial and *Legionella* control in the home. Those that did know gave answers as to why including “safety-burning,” “kids showering themselves,” “cost,” and personal preference to “hot showers”. No answers gave any indication that the temperature was set to reduce *Legionella* or microbial contamination.

## 3. Discussion

This study demonstrates that domestic household showers located in South Australia are a significant source of *Legionella* spp. and *L. pneumophila.* These findings support a previous study conducted in the UK by Collins et al. [5] that detected *Legionella* spp. and *L. pneumophila* in household showers located in South England and a recent study that detected *Legionella* along the South Australian municipal potable water distribution pipeline [11]. This study used qPCR and found that 74.6% and 64.2% of showers positive for *Legionella* spp. and *L. pneumophila*, respectively. This is significantly higher than the findings of the UK study, which reported 31% of showers to be positive for *Legionella* spp. using qPCR and only 6% positive using culture methods of detection [5]. This may possibly be linked to climatic differences in South Australia [4]. The most significant variables associated with increased *Legionella* concentrations found in this study were temperature and frequency of shower use, followed by the age of the house.

The hot water delivery temperature of shower taps ranged between 34 and 68 °C, with higher *Legionella* spp. concentrations statistically significantly associated with lower temperatures (*p* = 0.000). It is advised by the World Health Organization that hot water systems should ideally maintain a temperature above 50 °C to reduce microbial colonization [7] and previous studies have also demonstrated that buildings with hot water systems set under 60 °C were more likely to harbor Legionella [12]. The mean maximum water temperature recorded in this study was below 50 °C which could explain the high incidence of *Legionella* positive samples. These finding are supported by previous study conducted in Germany that identified temperature of hot water to be the most important determinant for *Legionella* growth in domestic residences [13]. Interestingly, the UK study did not find a statistically significant association between the presence of *Legionella* spp. and water temperature [5].

A statistically significant association between *Legionella* spp. concentrations and frequency of shower use (*p* = 0.000) was found. Higher concentrations of *Legionella* spp. were found in showers that were used on average less than once per month than those used once per day or more. This indicates that increased shower use is potentially protective against *Legionella* positivity. These findings are consistent with the current understanding that stagnant or low turnover of hot water are recognized as risk factors for *Legionella* colonization and proliferation [7], and also support the findings of the UK study [5].

Property and hot water system age have often been found to influence the occurrence of *Legionella* in showers [5,14]. This study found a statistically significant association between the age of house and *Legionella* spp. concentration (*p* = 0.037). Older houses were generally considered to harbor greater amounts of *Legionella* due to older infrastructure and more time to enable biofilm formation [5,13]. In this study, the highest concentrations of *Legionella* spp. were found in homes 10–14 years old and the lowest concentrations were seen in homes less than 5 years of old, which could be attributed to limited time for biofilm formation and colonization. This supports the findings of the UK study that found *Legionella* spp. was associated with the age of the property (*p* = 0.02), with newer houses associated with lower incidence of *Legionella* spp., followed by a steady increase in *Legionella* numbers until a decrease was observed for properties over 30 years old. In this South Australian study, houses over 30 years old were not identified; however, there was a large variation seen in houses over 20 years and a decrease in the mean concentration compared with houses 10–14 years old. It is difficult to determine possible factors related to higher positivity in relatively young properties (10–14 years old); however, differences in plumbing material (i.e., copper or PEX piping) have previously been suggested to influence *Legionella* colonization [5].

In this study, the type of hot water system was not statistically significantly associated with increased *Legionella* concentrations. Instantaneous hot water systems are generally considered a lower risk for *Legionella* contamination as they produce hot water instantaneously without storage [5,13,15]. However, this study found 74.6% of showers were positive for *Legionella* spp. and 64% of positive samples were from instantaneous systems, although this association was not statistically significant. The configuration of instantaneous water systems is such that temperatures above 50 °C are rarely achieved and residence time of water at elevated temperatures is minimal. This may negate the effects of temperature in controlling legionella and other bacteria entering the building’s water system. In turn, this may permit direct colonization of outlets.

The lack of public awareness regarding the risks associated with domestic hot water system identified in this study raises concerns. This is the first time in Australia that public perception regarding *Legionella* risks in the home has been quantified, and there is an apparent need for simple public health advice such as increasing hot water temperature or running showers every week to reduce the risk of a *Legionella* contamination. This is particularly significant for the elderly or immunocompromised who are most vulnerable to *Legionella* infection.

Whilst this study has demonstrated *Legionella* contamination is common in South Australian household showers, these sources are not routinely sampled during sporadic Legionnaires’ disease investigations. As a consequence, clinical significance and evidence to indicate a public health burden is yet to be determined. It is also important to note that although *Legionella* were detected in over two-thirds of the samples collected, the detection method does not differentiate between viable and non-viable bacteria. There is also no established equivalent value for the detection of *Legionella* using qPCR, and we are unable to align our results to current legislation to determine what showers may pose a public health threat. This is also highlighted by a UK study investigating the presence of *Legionella* contaminated aerosols from showers known to be positive for *Legionella*, which failed to detect viable *Legionella* using culture. However, the study did detect *Legionella* DNA in the aerosols using qPCR [5], and as such the negative culture results could be attributed to the presence of viable but non-culturable cells contained within the aerosols [16]. There is a need for further research to explore the role of domestic shower aerosols as a potential route of transmission for Legionnaires’ disease.

## 4. Materials and Methods

### 4.1. Sampling Sites

The study was approved by Flinders University Social and Behavioral Research Ethics Committee (SBREC No. 7291) in accordance with the National Statement on Ethical Conduct in Human Research (NSECHR). Households approached to participate in the study were randomly chosen based on the criteria that they were located within metropolitan Adelaide. Initial contact with homeowners was through direct contact (knocking on doors). The decision to participate in the study was voluntary. All data were geocoded and stored anonymously. From August 2016 until July 2018, a total of 68 showers from 50 households across metropolitan Adelaide were sampled.

### 4.2. Data Collection

Water samples were collected by researchers using a sterile 1000 mL wide-mouthed plastic collection bottle. Collection involved turning the hot water tap on to capture the first liter of water from the shower head (in the case of a mixer tap, it was set to the hottest setting before opening the tap). The bottle was then sealed, transported on ice to the laboratory and refrigerated within 4 h. Samples were then processed within 24 h. The hot water temperature was measured by researchers by allowing the hot water to flow for approximately 2 min, until the maximum temperature at the shower outlet was reached. This was then measured using a thermometer and recorded.

Surveys (Supplementary 1) were completed by the household occupant to determine the type of hot water system (gas, electric or solar), hot water supply (instantaneous or stored), occupant knowledge of the temperature setting (°C), age of hot water system, age of house, number of showers and shower use frequency. The results of the survey were used to establish relationships between *Legionella* concentration and the various factors explored in the survey questions.

### 4.3. DNA Extraction

*Legionella* DNA extraction was conducted using the BIO-RAD Aquadien^TM^ Bacterial DNA Extraction and Purification Kit following manufacturer’s instructions. DNA was extracted from the 1 L water sample providing a final volume of 100 µL of DNA extract (Bio-Rad Laboratories, Inc., NSW, Australia). 

### 4.4. Detection of Legionella by qPCR

*Legionella* spp. and *L. pneumophila* were enumerated using a qPCR method previously described by Giglio et al. [17] using SYTO9 as the intercalating fluorescent dye and their respective primers JFP/JRP and Mip99f/Mip213R. The 25 µL reaction volume contained 1× PCR buffer (Invitrogen), 2.5 mM MgCl_2_ (Invitrogen), 2.5 mM SYTO9 fluorescent dye (Invitrogen), 0.2 mM deoxynucleoside triphosphate mix (Invitrogen), I U platinum Taq DNA polymerase (Invitrogen), 5 µL template DNA and their respective primers: 0.3 µM JFP primer (5′-AGGGTTGATAGGTTAAGAGC-3′) and 0.3 µM JRP primer (5′-CCAACAGCTAGTTGACATCG-3′) (*Legionella* spp.); or 0.5 µM mip99F primer (5′-TGTCTTATAGCATTGGTGCC-3′) and 0.5 µM mip213R primer (5′-CAATTGAGCGCCACTCATAG-3′) (*L. pneumophila*).

All qPCR reactions were carried out in a RotorGene 3000 (Corbett Research, Sydney, Australia). Cycling conditions involved an initial hold at 95 °C for 5 min, followed by 40 cycles at 94 °C for 20 s, 20 cycles at 60 °C for 20 s and 40 cycles at 72 °C for 25 s. Data acquisition was attained at 72 °C on the 6-carboxyfluorescein channel (excitation at 470 nm, detection at 510 nm) at a gain of 5. Melt curve data were acquired on this channel at gains of 2 and 5 using a ramping rate of 1 °C over 60 s from 75 to 95 °C. For each reaction, the melt curve was analyzed and a positive *Legionella* spp. or *L. pneumophila* was confirmed with a melting temperature (T*_m_*) of 88 ± 1 °C and 82.5 ± 1 °C, respectively. The limit of detection for both *Legionella* spp. and *L. pneumophila* and was 2.5 copies/mL [11].

To determine the presence of environmental inhibitors in the neat DNA extract, each sample was diluted 1:10 using nuclease-free distilled water. Dilution of the DNA sample helps to reduce the inhibitor concentration and enhance qPCR efficiency. Previous studies have shown this method to be an effective alternative to help exclude inhibition from environmental samples [11,18,19]. The diluted samples were amplified in triplicate under the same reagent and cycling conditions used for the enumeration of *Legionella* spp. and *L. pneumophila.* If the cycle threshold (CT) value for 1/10 dilution of DNA was less than approximately 3.3 (representing an approximately 1-log_10_ concentration value) (Livak 2001) than the pure DNA extract, it was assumed that inhibitors were present and the 1/10 dilution was used to calculate copies/mL [11].

### 4.5. Statistical Analysis

Statistical analysis of results was conducted using SPSS software with a level of significance set at *p* < 0.05. Normality was tested using the Kolmogorov–Smirnov test. A Spearman’s rho test was used to identify associations between *Legionella* spp. and *L. pneumophila* concentrations and different household/plumbing features identified in the survey. When determining statistical significance of the association with different variables any “don’t know” survey responses were removed.

## 5. Conclusions

This study has demonstrated that domestic showers are frequently colonized by *Legionella* spp. and *L. pneumophila* and should be considered as such during source investigations. Statistically significant associations were found between water temperature, frequency of use and age of house, and increased concentrations of *Legionella* spp. This suggests that increasing hot water temperature and running showers every week could potentially reduce the risk of *Legionella* contamination in the home. This information could be useful in managing the risk of Legionnaires’ disease within the community, which is particularly relevant given our aging population and increase in elderly individuals wishing to remain living independently in their own homes. The current lack of awareness regarding the potential risks associated with household showers demonstrates the need for public health campaigns to inform vulnerable populations of the steps they can take to reduce the risk of exposure to *Legionella*.

## Supplementary Materials

The following are available online at https://www.mdpi.com/2076-0817/8/1/27/s1. Materials S1: Survey questions.
