# Supplementary material for: Factors Influencing Legionella Contamination of Domestic Household Showers
Source: Pathogens. 2019 Feb 26;8(1):27. doi: 10.3390/pathogens8010027 (PMC6470800; doi:10.3390/pathogens8010027)
Supplement: Supplementary file 1 [file pathogens-08-00027-s001.pdf]

Dr. Harriet Whiley

GPO Box2100
Adelaide SA 5001

Tel: 08 7221 8580
Fax: 08 7221 8590

Email: Harriet.Whiley@flinders.edu.au

Survey questions

1) Do you know what type of hot water system you have?

- ☐ Gas hot water
- ☐ Electric
- ☐ Solar hot water
- ☐ Don't know

2) Is your heated water stored in a tank prior to use, or do you have an instantaneous hot water system?

- ☐ Stored in a tank
- ☐ Instantaneous
- ☐ Don't know

3) Do you know the temperature your hot water service is set at?

☐ Yes   If Yes, please go to question 3a).

☐ No   If no go to question 5.

What temperature or setting is your hot water system set at?

a) _____^{o}C
b) __________ (i.e. low / medium / high / setting 1 - 4 / other settings?)

☐ Did you or someone in your home set the hot water system temperature, or

☐ Was the temperature set-up by the person who installed the hot water system for you, or

☐ Was the temperature of the hot water system temperature already set when you moved into the house/dwelling?

☐ It is too long ago, I can't recall who set the temperature

4) If you, or someone in your home had a choice in deciding the setting the temperature on your hot water system was there a reason as to why that particular setting was chosen?

☐Yes. Please answer 4 a)

☐No. If no please go to Qu. 5

☐Don't know. If you don't know please answer Qu. 5

a)____

____

____

Dr. Harriet Whiley

GPO Box2100
Adelaide SA 5001

Tel: 08 7221 8580
Fax: 08 7221 8590

Email: Harriet.Whiley@flinders.edu.au

5) Approximately, how old is your hot water system?

- ☐ Less than 5 years old
- ☐ 5-9 years old
- ☐ 10-14 years old
- ☐ 15-20 years old
- ☐ More than 20 years old
- ☐ Don't know

6) Approximately, how old is your house

- ☐ Less than 5 years old
- ☐ 5-9 years old
- ☐ 10-14 years old
- ☐ 15-20 years old
- ☐ More than 20 years old
- ☐ Don't know

7) How many showers are there in the house?

- ☐ 1
- ☐ 2
- ☐ 3
- ☐ 4
- ☐ 5

Typically, how often does shower 1 get used?

- ☐ Less than once a month
- ☐ less than once a week
- ☐ once a week
- ☐ once a day
- ☐ twice a day
- ☐ more than twice a day

Typically, how often does shower 2 get used?

Dr. Harriet Whiley

GPO Box2100
Adelaide SA 5001

Tel: 08 7221 8580
Fax: 08 7221 8590

Email: Harriet.Whiley@flinders.edu.au

- ☐ Less than once a month
- ☐ less than once a week
- ☐ once a week
- ☐ once a day
- ☐ twice a day
- ☐ more than twice a day

Typically, how often does shower 3 get used?

- ☐ Less than once a month
- ☐ less than once a week
- ☐ once a week
- ☐ once a day
- ☐ twice a day
- ☐ more than twice a day

Typically, how often does shower 4 get used?

- ☐ Less than once a month
- ☐ less than once a week
- ☐ once a week
- ☐ once a day
- ☐ twice a day
- ☐ more than twice a day

Typically, how often does shower 5 get used?

- ☐ Less than once a month
- ☐ less than once a week
- ☐ once a week
- ☐ once a day
- ☐ twice a day
- ☐ more than twice a day
